# Sensitivity Analysis of Selected Parameters in the Order Picking Process Simulation Model, with Randomly Generated Orders

**DOI:** 10.3390/e22040423

**Published:** 2020-04-09

**Authors:** Mariusz Kostrzewski

**Affiliations:** Faculty of Transport, Warsaw University of Technology, Koszykowa 75, 00-662 Warszawa, Poland; mariusz.kostrzewski@pw.edu.pl; Tel.: +48-22-234-7337

**Keywords:** high-bay warehouse, simulation model, order picking process, pseudorandom number generator, PRNG, logistics, warehousing, discrete event simulation

## Abstract

Sensitivity analysis of selected parameters in simulation models of logistics facilities is one of the key aspects in functioning of self-conscious and efficient management. In order to develop simulation models adequate of real logistics facilities’ processes, it is important to input actual data connected to material flows on entry to models, whereas most models assume unified load units as default. To provide such data, pseudorandom number generators (PRNGs) are used. The original generator described in the paper was employed in order to generate picking lists for order picking process (OPP). This ensures building a hypothetical, yet close to reality process in terms of unpredictable customers’ orders. Models with applied PRNGs ensure more detailed and more understandable representation of OPPs in comparison to analytical models. Therefore, the author’s motivation was to present the original model as a tool for enterprises’ managers who might control OPP, devices and means of transport employed therein. The outcomes and implications of the contribution are connected to presentation of selected possibilities in OPP analyses, which might be developed and solved within the model. The presented model has some limitations. One of them is assumption that one mean of transport per one aisle is taken into consideration. Another limitation is the indirectly randomization of certain model’s parameters.

## 1. Introduction

The introduction of the paper consists of three interrelated parts. At first, the significance of the order picking process was described. Secondly, prior research on the order picking process was identified. Thirdly, comprehensive discussion on the use of simulation approach for order picking is provided and objectives of the paper are defined.

### 1.1. Significance of Order Picking Process

An order picking process is considered as one of the most important research interests in the field of internal logistics. This is due to the fact that an order picking process engages most of the resources of all processes taken in logistics facilities, as confirmed in Alicke et al. (2001) and Ulbrich et al. (2016) [1,2]. Moreover, this process is considered to be the most time consuming, as it is given in Lu et al. (2016) and Roodberger and de Koster (2001) [3,4], and at the same time highly cost consuming. Chiang et al. (2011) in [5] estimated the cost of order picking—the authors of this study stated that the costs incurred during order picking processes vary from 55% to 75% of total cost for all logistics processes in logistics facilities (also in Kostrzewski 2014 and Drury 1988 [6,7]). In turn, Gałązka and Jakubiak (2010) [8] reported that these costs are estimated in the range of 55%–65% in comparison to the mentioned total cost. Other authors estimated the costs of order picking compared to a total operating cost in a warehouse at the level of 55% [9,10,11,12], 60% [7], 65% [13] and even 60%–70% according to [14]. These values are within the ranges mentioned above. On the other hand, Daly (1993) in [15] stated that the order picking process consumes 60% of labor intensity out of all processes in a logistics facility, such as a warehouse. The order picking process was also analyzed as the costliest warehouse activity in Marchet et al. (2015) [16] According to the research data stated above, over the years, the costs incurred during order picking process still fluctuate around half to three quarters of the total operating costs incurred for all logistics processes occurring in a warehouse (the quoted data date from the second half of 80s of the previous century—[7], and the last study cited in this paper that concerns of such extreme values was published in 2011—[5]). On the one hand, it could be argued that either the reduction of these costs is difficult in actual conditions, or that the changes proposed by various researchers are not implemented. On the other hand, further research on this subject matter is necessary, with the aim of the continuous and multi-faceted improvement of said process, particularly given that, according to Wruck et al. (2017) [17] (quoted in van Gils et al. 2018 [18]), high labor cost in the case of order picking processes may be treated as one of many results for the underperformance of logistics systems and as a consequence may lead to customers’ demands not being met.

### 1.2. Prior Research on Order Picking Process

The thematic scope of scientifically compiled studies on the order picking process is very broad. Various researchers deal with studies of the mentioned process connected to e.g., strategies used to achieve an efficient order picking process (e.g., [8,19]), cost estimation based on highly detailed and hierarchical analytical or numerical models, and the reduction of the duration of these processes (or indirectly, reduction of the travel distance between picking points, as in: [3,20,21,22]). The optimization or sub-optimization of the order picking process, achieved through the use of accurate methods of operational research or as a result of the implementation of heuristic methods, is also one of the topics that researchers take into consideration in the scientific literature. Numerous publications deal with issues related to analyses of logistics and transport processes in logistics facilities such as warehouses. For example, in [23], Chew and Tang (1999) analyzed time of freight transport process in a warehouse. They, as many other researchers, also took into consideration an order picking process. They applied queue theory and suggested arrangement of picking area in such manner, so that load units’ allocation was arranged for customers with similar expectations for ordered products. Transformations of the proposed model, designed for a single down-aisle, were presented in Le-Duc and de Koster (2007) [24]. The authors implemented this model for the full route of means of transport, as part of a picking operation. In [23], Chew and Tang (1999) examined a monobloc warehouse, i.e., without taking into account a transverse corridor between two blocks consisting of rows of racks situated in parallel to each other. In [24], Le-Duc and de Koster (2007) introduced a transverse corridor and analyzed two-block type of warehouse. An interesting new layout problem called “discrete cross aisle warehouse design” is addressed in Öztürkog et al. (2019) [25]—authors developed a new warehouse layout that ensures the travel distance reduction for an order picking process. Authors proved, that in comparison to traditional two-block layouts, the new one provides 7% savings in travelling length, on average, in the case of the order picking process. The issues of estimation of order picking process time and the attempt to reduce it, apart from the aforementioned research, were dealt with in Gibson and Sharp (1992) [26], where authors took into account the ABC analysis as one of the stock management methods applied in a warehouse. Many other authors attempted to reduce order picking process time, such as Davarzani and Norrman (2015) [27] and Tompkins et al. (2010) [9]. Davarzani and Norrman (2015) in [27] presented a wide range of order picking solutions, investigated both in practice and theory, and they mentioned researchers who have shown cost and time efficiency of employing these particular solutions for warehousing technologies (Chow et al. 2006 [28]). Tompkins et al. (2010) defined order picking costs as 55% of total warehouse operating costs in [9]. Numerous other researchers also investigated these areas of interests. Therefore, it can be stated that the subject matter of the order picking process continues to be an issue of high interest. It is because this topic is still not sufficiently researched. Order picking is one of the most complicated processes occurring in logistics facilities, and that is why it requires ongoing research, continuous process optimization, etc. Moreover, it is a very complex process. It is not without reason that a vast amount of research has been devoted to this aspect and the topic is still being exploited, even if only a few of articles representing this subject are mentioned in this contribution (a more extensive discussion of this subject matter is provided in the next paragraph). In addition, it has been recognized in House and Karrenbauer (1978) [29] (p. 192), that time is the only variable that can be used to describe a logistics system. The authors added that in the past, logisticians have described systems as a function of distance, but over time it became obvious that distances were attributes not homogeneous enough within the logistics networks. The author of this paper is not of the opinion that time should be considered as the only variable that can serve to describe the logistics system, but as one of the more important ones. However, the importance of this kind of variable is proven by van Gils et al. (2018) [18], who stated that the order picking process time is a performance indicator in more than 73% of references analyzed by themselves, overcoming indicators from other groups such as cost, productivity and service (according to van Gils et al. (2018) [18], this classification was distinguished by Staudt et al. 2015 [30]).

As the classical way of order picking modeling shows (Fijałkowski 1995 [31]), calculations obtained with the use of analytical models, and in particular analytical models concerning order picking processes, are most often carried out on the basis of a predetermined structure of orders (averaged quantities of lines in a picking list and quantities of items to pick per one line are treated as non-variant, constant values, unique and unambiguous in the scale of the whole picking system). Yet, in real conditions, there is a significant variation in both the quantities of items per line in each order (further in the paper this parameter is noted as $p_{ij}$—each time, subscript *j* is designated to a consecutive number of experiment in a sample, and subscript *i* is designated to the quantities of items), and the varied range of items to pick (later in the paper this parameter is noted as $w_{j}$). In comparison to the referenced simulation models the author’s approach to generation of order picking lists is more detailed and thus more adequate to actual conditions. Three main ways of picking lists generation were identified in the literature: generalized random picking lists, uniform distribution picking lists and picking list based on historic data. The list of research papers in which these different approaches were identified is synthetized in Table 1 (other ways of picking lists generation were also mentioned in the last row of Table 1). The performed analysis shows, that the application of generalized random picking lists (e.g., based on the Monte Carlo simulation method) was evidently applying pseudorandom number generators (PRNG), or there is no clear indication of how a particular random picking list was achieved. This means that, in the case of several elaborations mentioned in Table 1, ‘random’ is not specified as such, therefore the author of this paper assumes the application of particular PRNG built-in software in the referred research. When uniform distribution picking lists were identified in literature, a picking list with such uniform distribution was mentioned in the certain research. The last group of historical picking lists; particular picking lists were compiled on the basis of actual data collected from entrepreneurs, who, the author assumes, agreed to collaborate with mentioned researchers during their research. Table 1 clarifies three different approaches to generating order picking lists. Previous research showed that for uniform distribution generators, the results are repetitive [32]. Meanwhile, Karkula (2013) [33] (p. 70) stated that uniform distribution is applied as default to generate pseudorandom numbers, which is key information resulting from the literature review.

As a result of the literature review presented in Table 1, it can be stated that the majority of the reviewed research was applied with the use of a unified distribution picking list (as was speculated in [33]), together with a generalized random picking list. Numerous researchers mentioned that random numbers are used for picking lists generated in their research. Those who did not apply either probability distribution or the pseudorandom numbers generator applied historical data obtained from real-world logistics facilities. However, it is worth pointing out that it is not always possible to obtain data from real-world systems (which mostly results in trade secrets’ procedures). Therefore, the author of the paper, in his research, decided to face the up-to-date problem of order picking, namely the development of a hypothetical simulation model, in order to describe it in the most realistic way possible, with taking into consideration the actual, unpredictable size of picking orders.

In the paper, as a result of employing simulation methods to model order picking processes, the interdependence of physical objects (infrastructural and moveable) in a particular warehouse system is presented. In addition, the sensitivity of this system’s operating parameters to changes within the system is considered. Moreover, the impact of these changes on the achievement of objectives of the system operations is presented. These changes are understood in particular as: means of transport failures or stoppages, human factor problems, variability of picking lists, etc. The author assumed that this type of sensitivity was assessed on the basis of values obtained during the order picking process duration analysis.

As mentioned above, the research discussed in this paper was carried out using simulation methods. Discussion on the potential of using simulation methods and tools to build simulation models can be found in [32,33,100,101], among many others. Therefore, discussion on the mentioned potential and tools is omitted in this paper, especially since it is a matter of modeling and simulation theory, of which the description would require a very long dispute. It is not without a reason that books devoted to the theory of simulation, which is implemented into the research in this article, are quoted here [32,33,101], indicating only a few of them (since there are numerous publications on the topic). It is important, however, to emphasize the fact that the simulation model discussed in this paper is a stochastic model. In case of stochastic models, the random variables that influence mechanisms involved in the implementation of logistics processes are of great importance. It is not possible to detect a linear pattern of event occurrences in the processes in this particular simulation model. An important element in the construction of such a model is the selection of a suitable generator of pseudorandom values. The choice of the PRNG, which was used in the presented research, was preceded by numerous experiments with the application of different PRNGs, based on selected probabilistic distributions (mentioned in Table 1). These experiments were discussed in previous publications [32,102,103].

### 1.3. Objectives of the Research

There are several objectives of the research and the paper that correspond to each other. First of all, it is the literature review which aims at determining the importance of order picking processes’ consideration, in particular the implementation of these processes in the form of simulation models of hypothetical warehouses, reflecting real-world warehouses. Secondly, the research contributes to verifying the applicability of a selected pseudorandom number generator (PRNG) in the simulation model, in order to reflect real-world processes in said simulation model. To be more specific, PRNG is used to give a stochastic character to the processes that are reflected in the form of a simulation model for analyses of the order picking process in a high-bay warehouse. A discussion on PRNGs can be found e.g., in: [101] (according to best knowledge of this paper’s author, PRNGs in connection to order picking process analyses and research are not often published—the only paper found in the Science Direct scientific database was the paper [102]—accessed in February 2020). The stochastic character of several variables used in the simulation model in this paper is given in each subsequent simulation experiment. The above-mentioned variability is subjected to order picking parameters, including: the number of lines (rows) in the picking orders’ list (in short: picking list) and the quantity of items (products of one type) to pick. Thirdly, selected parameters that are generated based on a particular PRNG are analyzed in the aspect of sensitivity of selected parameters of a simulation model (these selected parameters are connected to the randomness of a simulation model). Therefore, the whole research is guided by one generalized, basic objective of research, which is stated as follows: modelling of an order picking process in a hypothetical logistics system that reflect real-world ones.

The subsequent sections discuss the following topics. Section 2 discusses the simulation model based on an analytical (conceptual) model. Author of this paper explains how the individual components of an analytical model are reflected in a simulation model. Subsequently, a problem of verification and validation of a simulation model used in the research is described in Section 3. PRNGs are applied in order to emphasize the stochastic nature of a simulation model, dedicated to the analysis of an order picking process in a high-bay warehouse. Stochastic character is assigned to variables in each subsequent simulation experiment. These variables include quantities of items to pick and numbers of rows in picking lists, per se. Thus, Section 5 deals with the use of the best matched PRNG in the analysis of sensitivity of operating parameters in the hypothetical logistics system which is under consideration herein. The last section consists of a summary and suggestions for further research on discussed issues.

## 2. Conceptual and Simulation Models

Simulation models are used when it is not possible, or it would be difficult to obtain, an analytical solution to a certain investigated problem, or when a comparison of analytical and simulation solutions is recommended. The latter applies to this research. The procedure for the use of simulation methods in warehouse design and research is described in [32,104], and therefore it is omitted in this contribution. The basic contents of simulation theory are also omitted. These include the main simulation types, advantages and disadvantages of simulation methods’ (and models’) implementation and other aspects of simulation modeling, which can be found e.g., in [32,33,104]. The aforementioned aspects of simulation methods and theory are omitted in the paper due to the fact that these are topics which are already extensively described in literature.

The simulation model discussed in this paper was developed in accordance with the Discrete Event System Specification (DEVS) within the Plant Simulation software. The DEVS structure is defined in [101]. The means of transport that is used here is EKX_515k, an electric three-way stock-picker lift-truck [105]. Therefore, the order picking system researched here deals with a picker-to-parts type of system. A picker-to-parts system is understood as one in which order picker travels along an aisle in order to retrieve parts/products/units/entities. In over 80% of logistics facilities in Western Europe, where order picking processes take place, these processes are carried out by employees [12]; meanwhile, the scientific literature is more focused on picking with the use of AS/RS (the acronym stands for an automated storage and retrieval system), AVS/RS (the acronym stands for an autonomous vehicle storage and retrieval system) systems [12]. The latter kind of system is used in large majority of logistics facilities in Western Europe [12,16]—therefore, it also needs to be properly analyzed by researchers.

The simulation model that was built for the research allows one to generate 100 picking lists per each simulation model execution. The number of lines in each picking order reaches the scope of $w_{j}$ = 1 ÷ 10, and the quantities of items to pick (per line) are within the scope of $p_{ij}$ = 1 ÷ 10. In general, in comparison to literature, e.g., [74], the number of customer orders is fixed to 100, whereupon the quantities of items per order is distributed uniformly over the set {1, 2, …, 10}. Picking lists are generated as a result of the initiation of the Generate orders procedure. The procedure inputs picking lists into the Orders table, which contains all of the orders, the quantities of the items ($p_{ij}$) per line in each of the hundred orders, and the varied range of items ($w_{j}$). In order to generate quantities of items per single order line, the adequate transformation of the logistics map (the origins of which are in chaos theory) into PRNG was proposed. The construction of the simulation model is preceded by a description of a conceptual model, which is based on aforementioned analytical relationships (analytical model).

The core equation of the simulation model is converted and redefined the logistic differential equation, which is discussed in [32]. The elements of both analytical (or conceptual, which is based on the analytical model) and simulation models are presented and discussed in the paper. A description of the models requires an input of the following parameters. Each time, subscript/parameter *j* is designated to the consecutive number of an experiment in a sample, and subscript/parameter *i* is designated to the quantities of items. Some elements of the investigated analytical model were presented in [104] and the full model was given in [32]. Since the language of these two publications is Polish, the author considers them worth quoting and briefly describing in this paper.

In this paper, the problem is discussed on the example of a pseudorandom number generator, in which a discrete equivalent of logistic differential equation is applied. The logistic differential equation is known as the simplest model of chaos. In Gutenbaum (2003) [106] (p. 96), it was found that relatively simple, strongly nonlinear deterministic differential equations are good models of some complex dynamic processes. In addition, it was noted that this type of mathematical equation, within a certain range of initial conditions and coefficients, allows one to obtain solutions with features of random processes. Processes of this type are qualified as chaos, in fact deterministic chaos, and their scientific basis derives from hydrodynamic flow studies in meteorology; Lorenz (1963) [107]. Deterministic chaos usually refers to nonlinear deterministic differential equations describing dynamic systems. It is understood as an irregular motion derived from a nonlinear system, in which dynamics uniquely determine the evolution of a system in time if the system’s history is known, and the real causes of irregularities are the characteristics of nonlinear systems, which are the exponential divergence of initially close trajectories in a limited area of a phase space; Schuster (1993) [108].

The solution of the logistic differential equation resembles a random sequence which can be used successfully in order to generate random numbers. The equation is defined in [106] (p. 96), and given here under discussion is Equation (1).

(1) $$y_{j}=\alpha \cdot \left(a-y_{j-1}\right)\cdot y_{j-1};\;j=\bar{1,J}$$

where: α—coefficient of growth that vary as α∈0;4, a—state of saturation, $y_{j}$—value at *j*th iteration step (e.g., population increase in biology or material flow increase in logistics)—it is a number between zero and one that represents the ratio of current size of the material flow to the maximum possible volume of material flow. This equation, also known as logistic mapping, is a modification of the linear function $y_{j}=\alpha \cdot y_{j-1}$. Element $\left(a-y_{j-1}\right)$ maintains consecutive values in the iteration of a *j*th step in the form of a decimal fraction in the range (0;1〉 (when a = 1 then $y_{j}\in (0;1\rangle$). This equation is called an iterative (iterated function), since a result from a previous step is necessary to obtain a result in a following step. The logistic representation is thus characterized by a feedback loop. Its differential form can be described by the differential equation of the form $\dot{y}=f\left(y\right)$, given here as Equation (2).

(2) $$\frac{dy}{dt}=\alpha \cdot \left(a-y\right)\cdot y$$

As a result of using Equation (1) for successive values of the growth coefficient α, attractors are obtained, which draw subsequent elements of the *y_j_* series (as the value of α attractors increases more and more often, they accept bifurcation values). At *α* < 1, the attractor is a point and is equal to 0. At 1 < *α* < 3, the attractor is a point and is equal to 1−1α. In turn, for α = 3, the fixed point loses stability and the bifurcation of the value *y_j_* starts, while above orbit loses stability, and for higher values of α than $\alpha =1+\sqrt{6}$, bifurcation is multiplied. In the case of the value α = 4, the structure becomes chaotic, and the so-called strange attractor covers the whole range of values $y_{j}\in (0;1\rangle$, which strengthens the randomness of the obtained results. Therefore, for the purposes of this research, α = 4 was assumed. To adjust the form of the equation to this research, it is modified in accordance with the relationship indicated as the converted and redefined logistic differential equation given later in the paper.

The author assumes that W (Equation (3)) is the set of numbers of rows in the picking list, where *j* is the number of experiments in the one hundred-element sample test, and $w_{j}$ is the number of rows in the picking list in the case of a *j*th experiment in the sample (one experiment is understood here as the realization of an order picking process for one picking list). In the simulation model, parameter $w_{j}$ is the cardinality of picked items $p_{ij}$ of *i*th type in *j*th experiment, in cases where values of $p_{ij}$ are greater than zero.

(3) $$W=\left\{w_{1},\ldots ,w_{j},\ldots ,w_{J}\right\};\;w_{j}=count\left(p_{ij}|p_{ij}>0\right);\;j=\bar{1,J};\;i=\bar{1,I};\;w_{j}\in N^{+}+\left\{0\right\}$$

P is the set of quantities of items to pick. The notation for this set is given as Equation (4). It is a stochastic value $X\left(e_{p}\right)$, generated according to the PRNG, described in detail in the following section. In the case of implementation of the analytical model, the average value of all quantities of picked items in *j*th experiment $\bar{p_{j}}$ should be used, as in Equation (5), where $\bar{p_{j}}$ is an average value of $p_{ij}$. In the implementation of the simulation model, it is not necessary to use $\bar{p_{j}}$, since construction of the simulation model allows the use of plain $p_{ij}$.

(4) $$P=\left\{p_{11},\ldots ,p_{ij},\ldots ,p_{IJ}\right\};\;p_{ij}=X\left(e_{p}\right);\;j=\bar{1,J};\;i=\bar{1,I};\;p_{ij}\in N^{+}+\left\{0\right\}$$

(5) $$\bar{p_{j}}=E\left(p_{11},\ldots ,p_{ij},\ldots ,p_{IJ}\right);\;j=\bar{1,J};\;i=\bar{1,I}$$

In the simulation model, the transformation of the logistics map (defined in [32]) is used as PRNG for $p_{ij}$ estimation (Equation (4)). This transformation is presented in the paper as converted and redefined logistic differential equation—Equation (6). Parameter of type $p_{ij}^{*}$ accepts decimal values in Equation (6) and moreover $p_{ij}^{*}<1$, thus a multiplier by 10 is entered to obtain $p_{ij}$ according to the structure of the experiment, that is: $p_{ij}\in \langle 0;10\rangle$, $p_{ij}\in N^{+}+\left\{0\right\}$. It should be stressed that the random numbers $p_{ij}^{*}$ are merged into a fraction located in the interval between 0 and 1. In Karkula (2013) [33] (p. 70), it is stated that uniform distribution is the basic distribution used to generate pseudorandom numbers of other distributions (the algorithm for generating an uniform random number between 0 and 1 is described in L’Ecuyer (1988) [109]).

(6) $$p_{ij}=X\left(e_{p}\right)=⎡10\cdot 4\cdot \left(1-p_{\left(i-1\right)\left(j-1\right)}^{*}\right)\cdot p_{\left(i-1\right)\left(j-1\right)}^{*}⎤;\;p_{11}^{*}\in (0;1\rangle ;\;j=\bar{1,J};\;i=\bar{1,I}$$

The set $T_{EX}$ of order picking process times is given as Equation (7). It is a set of elements $t(j,w_{j},\bar{p_{j}},EX)$ described on the set of positive real numbers, where *EX* means that the analytical model is implemented in MS Excel software. This model is based on the classical approach of order picking process time calculation [31], given as Equation (8). The parameters used in this equation are as follows:

- mean value of lift-truck acceleration or stop, *A*;
- length of a rack in warehouse, *L*;
- mean time of lift-truck driving forward or backward (with lowered cabin and forks), $F_{1}$;
- mean time of lift-truck driving forward or backward (with lifted cabin and forks), $F_{3}$;
- rack height (from the ground to the bottom of the highest rack storey), H;
- free lift of forks, $h_{2}$;
- medium value of load unit lifting up time, U;
- medium value of load unit lowering time, D;
- mean time of lift-truck fork ejection or rotation, *N*;
- time of picking list reading by employee, $t_{pro}$;
- time of reading the next row in a picking list, $t_{ro}$;
- time of single item picking, $t_{pl}$.
  (7) $$\begin{array}{c} T_{EX}=\left\{t\left(1,w_{1},\bar{p_{1}},EX\right),\ldots ,t\left(j,w_{j},\bar{p_{j}},EX\right),\ldots ,t\left(J,w_{J},\bar{p_{J}},EX\right)\right\};\;j=\bar{1,J};\\ t\left(j,w_{j},\bar{p_{j}},EX\right)\in T_{EX}\to {ℜ}^{+} \end{array}$$
  (8) $$\begin{array}{c} \forall p_{ij}\in P\;\forall w_{j}\in W\;\forall j\in \bar{1,J}\;\;\forall i\in \bar{1,I}:\;t\left(j,w_{j},\bar{p_{j}},EX\right)\\ =\left(w_{j}+1\right)\cdot A+L\cdot \left(F_{1}+F_{3}\right)+\left(n\cdot H+h_{2}\right)\cdot \left(U+D\right)+6\cdot N+t_{pro}+w_{j}\cdot \left(t_{ro}+\bar{p_{j}}\cdot t_{pl}\right) \end{array}$$

The set $T_{PS}$ of order picking process time is given here as Equation (9). It is a set of elements $t\left(j,w_{j},p_{1j},\ldots ,p_{ij},\ldots ,p_{Ij},f,MTTR,PS\right)$, described on a set of positive real numbers, where: $p_{1j},\ldots ,p_{ij},\ldots ,p_{Ij}$ correspond to quantities of items of *i*th types picked in *j*th experiment. The parameter *f* is an estimated percentage of simulation time, during which a mean of transport failure may occur, or a time when a mean of transport is fully incapable for work. The parameter *MTTR* stands for a mean time to repair (an average time from a failure moment to a repair completion of a defective device of a mean of transport or downtime, that is not necessarily caused by a system’s failure), when means of transport serving order picking processes are out-of-order. *PS* means that this equation is implemented in the Plant Simulation software. Stochastic differentiation of parameters *f* and *MTTR* is beneficial to the simulation model because these make the model more realistic, contrary to analytical calculations in their pure form.

(9) $$\begin{array}{c} T_{PS}=\left\{\begin{array}{l} t\left(j,w_{j},p_{11},\ldots ,p_{i1},\ldots ,p_{I1},f,MTTR,PS\right),\ldots ,\\ t\left(j,w_{j},p_{1j},\ldots ,p_{ij},\ldots ,p_{Ij},f,MTTR,PS\right),\ldots ,\\ t\left(J,w_{J},p_{1J},\ldots ,p_{iJ},\ldots ,p_{IJ},f,MTTR,PS\right) \end{array}\right\};\;j=\bar{1,J};\;i=\bar{1,I};\;\\ t\left(j,w_{j},p_{1j},\ldots ,p_{ij},\ldots ,p_{Ij},f,MTTR,PS\right)\in T_{PS}\to {ℜ}^{+} \end{array}$$

This part of the model is based on the classic approach to the order picking process time calculation [31], however it takes into account the complete information on orders in the randomly generated picking list, not just averaged values of lines’ numbers in the picking list or quantities of items to pick (per one line). This model also provides randomly generated downtimes in the work of means of transport. This manner of calculation is given in the generalized form in Equation (10).

(10) $$\forall p_{ij}\in P\;\forall w_{j}\in W\;\forall j\in \bar{1,J}\;\forall i\in \bar{1,I}:\;t\left(j,w_{j},p_{1j},\ldots ,p_{ij},\ldots ,p_{Ij},f,MTTR,PS\right)$$

In order to compare the analytical and simulation models, a goodness-of-fit test for two mean values comparison was used. For each hundred-element sequence of corresponding experiments, average order picking time values were computed for both types of the models. The average value of the order picking process time is defined and expressed as $\bar{t\left(EX\right)}$ in Equation (11), according to the conceptual (analytical) model. The other average value regarding the simulation model is given as Equation (14).

(11) t(EX)¯=E(t(j,wj,pj¯,EX))=(∑j=1Jt(j,wj,pj¯,EX))J; j=1,J¯

The dispersion of individual measurements around mean value for the conceptual model $s_{t\left(EX\right)}$ is calculated according to the Equation (12) and for the simulation model $s_{t\left(PS\right)}$ according to the Equation (15). The mean squared error of the mean value equations in the analytical and the simulation models, that indicate the degree of accuracy of the mean value, are $s_{\bar{t\left(EX\right)}}$ (Equation (13)) and $s_{\bar{t\left(PS\right)}}$ (Equation (16)), respectively.

(12) $$s_{t\left(EX\right)}={\left[\frac{1}{J-1}\sum_{j=1}^{J}{\left(t\left(j,w_{j},\bar{p_{j}},EX\right)-\bar{t\left(EX\right)}\right)}^{2}\right]}^{0.5};\;j=\bar{1,J}$$

(13) $$s_{\bar{t\left(EX\right)}}={\left[\frac{1}{J\left(J-1\right)}\sum_{j=1}^{J}{\left(t\left(j,w_{j},\bar{p_{j}},EX\right)-\bar{t\left(EX\right)}\right)}^{2}\right]}^{0.5};\;j=\bar{1,J}$$

(14) t(PS)¯=E(t(j,wj,p11,…,pi1,…,pI1,f,MTTR,PS),…,t(j,wj,p1j,…,pij,…,pIj,f,MTTR,PS),…,t(J,wJ,p1J,…,piJ,…,pIJ,f,MTTR,PS))=(∑j=1Jt(j,wj,p1j,…,pij,…,pIj,f,MTTR,PS))J; j=1,J¯; i=1,I¯

(15) $$s_{t\left(PS\right)}={\left[\frac{1}{J-1}\sum_{j=1}^{J}{\left(t\left(j,w_{j},p_{1j},\ldots ,p_{ij},\ldots ,p_{Ij},f,MTTR,PS\right)-\bar{t\left(PS\right)}\right)}^{2}\right]}^{0.5};\;j=\bar{1,J};\;i=\bar{1,I}$$

(16) $$s_{\bar{t\left(PS\right)}}={\left[\frac{1}{J\left(J-1\right)}\sum_{j=1}^{J}{\left(t\left(j,w_{j},p_{1j},\ldots ,p_{ij},\ldots ,p_{Ij},f,MTTR,PS\right)-\bar{t\left(PS\right)}\right)}^{2}\right]}^{0.5};\;j=\bar{1,J};\;i=\bar{1,I}$$

For both models, experiments were executed based on the following data:

- mean value of lift-truck acceleration or stop, *A* = 0.0475 [min] (value based on [31]);
- length of a rack in warehouse, *L* = 150 [m];
- mean time of lift-truck driving forward or backward (with lowered cabin and forks), *F*_1_ = 0.0079 [min/m] (the value of mean of transport velocity, i.e., *v* = 10.5 km/h, given in [105] has been converted to the *F*_1_ parameter, which is used in the analytical calculations; in turn, the velocity of the modeled mean of transport has been noted as *v_sym_* = 0.8547 [m/s], which is related to the simultaneous considerations on the mean of transport forward or backward movement with the lifted cabin and forks);
- mean time of lift-truck driving forward or backward (with lifted cabin and forks), *F*_3_ = 0.0116 [min/m] (due to lack of data in the catalog [105], the value is estimated on the basis of the analogy indicated in [31], i.e., from the *F*_1_/*F*_13_ ratio);
- rack height (from the ground to the bottom of the highest rack storey), *H* = 14.5 [m] (the adoption of this value is dictated by the fact that the lifting height in the catalog [105] is 14 570 [mm]);
- free lift of forks, *h*_2_ = 0.8 [m] (value based on [105]);
- medium value of load unit lifting up time, *U* = 0.0833 [min/m] (the lifting velocity *v_U_* = 0.2 [m/s] given in [105] is used to determine this value);
- medium value of load unit lowering time, *D* = 0.0417 [min/m] (the lowering velocity *v_D_* = 0.4 [m/s] given in [105] is used to determine this value);
- mean time of lift-truck fork ejection or rotation, *N* = 0.13 [min] (value based on [31]);
- time of picking list reading by employee, $t_{pro}$ = 0.0852 [min] (value based on [31]);
- time of reading the next row in a picking list, $t_{ro}$ = 0.118 [min] (value based on [31]);
- time of single item picking, $t_{pl}$ = 0.118 [min] (value based on [31]).

## 3. Verification and Validation of Simulation Model

Among the conditions, which enable the practical application of simulation models, validation and verification are mentioned [32]. Validation is only possible when the actual system was tested. The system presented in this paper is hypothetical, so it is only possible to verify the work of the simulation model and the verification without validation is accepted to be sufficient (this section is based on [32]).

For the purpose of verification, the values mentioned at the end of the previous section of this paper are implemented in conceptual (analytical) and simulation models. In addition, the estimated percentage of simulation time during which means of transport may be idle is assumed to be a random parameter *f* = *random* (0;0.1). The *MTTR* is assumed as a constant value and is equal to *MTTR* = 2 [min] in the case of means of transport implementation and *MTTR* = 1 [min] in the case of items picking and loading operations. Without saying, these times occur only in case of temporary damage, downtime or failure of implemented means of transport. Differently to the part of the research described in the following section, the PRNGs included in the software are used for the simulation model verification. In Plant Simulation, two PRNGs create a random integer, using the multiplicative linear congruential generator (MLCG), based on the algorithm given in [109].

As a result of Equation (11), the average order picking process time is computed, and its value is equal to $\bar{t\left(EX\right)}$ = 19.85 [min]. As part of the verification of the simulation model, 100 simulation scenarios are executed with identical, constant data, as for the analytical model. Average order picking process time obtained during simulations is equal to $\bar{t\left(PS\right)}$ = 19.16 [min] ($s_{t\left(PS\right)}$ = 0.30 [min], $s_{\bar{t\left(PS\right)}}$ = 0.03 [min]). The difference between values of parameters such as $\bar{t\left(EX\right)}$ and $\bar{t\left(PS\right)}$, which is equal to 3.5%, is negligible. This is due to the implementation of potential equipment failures and possible temporary downtimes in the process, connected to e.g., human behavior, physiological needs of lift trucks operators, etc.

In this research, the ordinary simulation lasted only 3 or 4 s (this corresponds to the process time which duration is about 27 h in the actual processes). The technical parameters of the computer used for experimentation were: *INTEL*^®^*Core*™ i3 CPU M370 @ 2.40 GHz, 4.00 GB RAM.

## 4. Discussion on Sensitivity Analysis of Selected Parameters in the Simulation Model

In applied sciences, sensitivity allows one to determine how large changes in the output of a system are caused by small changes in the reference signal at the input. Sensitive systems are characterized by very large changes on the output, in response to small changes at the input.

In order to analyze the sensitivity of selected parameters of the simulation model, parameters of f and *MTTR* are assumed to be subjected to changes.

**Figure 1:**
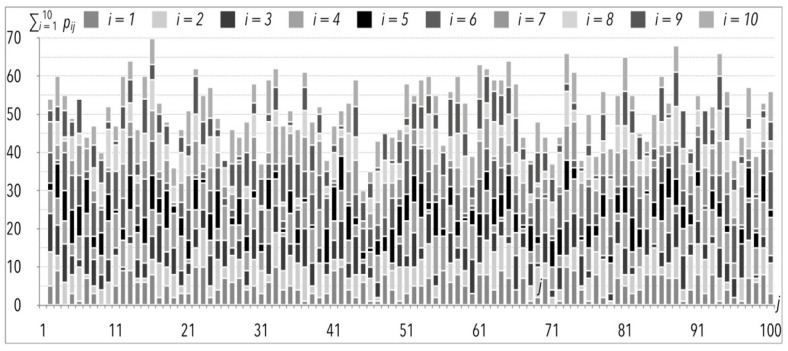
The changes in the order picking process time in case of the analyzed sample, with the indication of the number of items picked per order of *j*th number.

As the f coefficient is subjected to changes in subsequent experiments, from now on it is marked as f(k), where *k* is the subsequent simulation experiment. In [32], twelve simulation experiments were carried out, under which values of all parameters of the simulation model were not subjected to variability, except for the f(k) coefficient. In this paper, twenty-one experiments of such kind are presented. The mentioned constant values (not subjected to variability) are the same as in Section 2, and values of $p_{ij}$ are presented in Figure 1 and Table 2. It is assumed that in each *k* experiment, the increment Δf(k) would be equal to 5% in most of the cases. The only exception is the extreme value of 100%, which would mean that the system is completely suspended, therefore f(k=21) = 99%. The results of sensitivity analyses are presented in Table 3.

**Figure 2:**
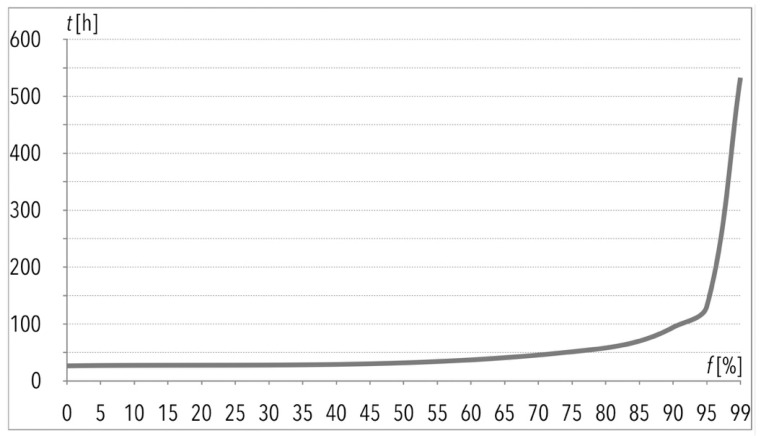
Execution of 100 picking lists for data sample, given in function f=f(k) in the case of total order picking process time (*t*).

**Figure 3:**
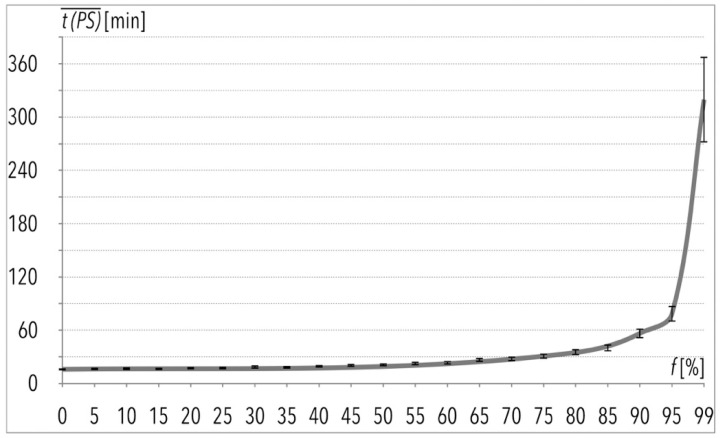
Execution of 100 picking lists for data sample, given in function f=f(k) in the case of the average value of order picking process time.

**Figure 4:**
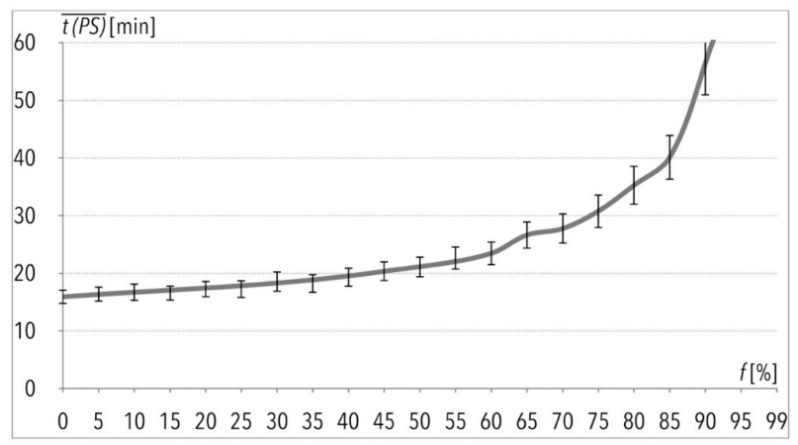
Execution of 100 picking lists per data sample, given in function f=f(k) in the case of the average value of order picking process time—part of the plot (the maximum value on $\bar{t\left(PS\right)}$ axis would be almost 60 min).

The total order picking process time as a result of the one hundred picking lists execution (given in Figure 1) is of particular interest to the author. This operation, without any disturbances i.e., f(k=1) = 0%, would take 26.5 h, and with 99% of disturbances, it would take more than twenty times longer (Table 3). Based on the mentioned data and taking limited assumptions into consideration, the author suggests that changes of the total order picking process time in function of f(k) are characterized by exponential growth, as can be observed in Figure 2. The same applies to the average order picking process time $\bar{t\left(PS\right)}$, given in the function of f(k) in Figure 3 and Figure 4.

The use of means of transport is reasonable at the maximum value of f(k=1) = 30% (empirically proven in [32]). This is due to the fact that the difference between the average order picking process time for the current and previous picking list in a given sample (e.g., absolute value from $\bar{t\left(PS\right)}|f\left(k=7\right)$ minus $\bar{t\left(PS\right)}|f\left(k=6\right)$ from Table 3) should not be lower than the degree of accuracy of the mean value (in reference to the example from the bracket, this is $s_{\bar{t\left(PS\right)}}|f=30\%$). The above results confirm the results of the study presented in [32]. For this paper, changes of the *MTTR* parameter are also introduced. Another 18 experiments are executed for the purpose described above, since now *k* is equal to $k=\bar{1,K}=\bar{22,39}$.

Table 4 shows that when f(k) is equal to, or less than 30%, process realization does not cause major problems, regardless of the *MTTR* value. Such a conclusion is drawn after familiarizing with $\bar{t\left(PS\right)}$ values. Taking into account the highest value of *MTTR* = 243 [min], i.e., c.a. four hours, it can be noticed that with two work shifts in the investigated logistics facility, this value does not exceed 30% of the time of means of transport incapacity, because a four-hour period is equal to 25% of the work shifts time. The shape of Table 3 has changed in comparison to Table 4, because in the case of *MTTR* analysis, it is also worth analyzing the means of transport workload in aspects of working time, waiting for the next order and downtime resulting from unforeseen circumstances (failure time). It is worth noting that the increase of *MTTR* values does not have as tremendous influence on the reduction of the means of transport working time as the f(k) values. In order to eliminate potential problems with process handling, a redundant replacement of means of transport or employees is made available. The values indicated in Table 4 and the above explanations confirm the results of earlier studies on the f(k) coefficient.

## 5. Conclusions

This paper considers important matters from the point of view of stochastic simulation models creation and experimentations employing them. Most of the analytical or simulation models assume unified load units for the whole system ([29]; e.g., in [110]). Moreover, simulation models ensure more detailed and more understandable representation of order picking processes and operations in comparison to analytical models [18]. This is a matter of high importance for warehouse managers, who can use simulation models as decision support tools, in order to design efficient logistics facilities such as warehouses, or in order to improve the existing, real-world facilities of this kind (by analyzing the interaction between operations and processes in a simulation mode without any risk of such analyses in real-world facilities). The model presented in the paper can be used especially by industries which deal with handling of multiple small units, in particular selected major industries such as: electronic industry, food industry, textile industry, automotive industry, chemical industry (pharmaceutical industry), steel industry and construction industry. With regard to the International Standard Industrial Classification of All Economic Activities [111], these industries would be focused on sections such as: Manufacturing (Section C: Division 10 Manufacture of food products, Division 11 Manufacture of beverages, Division 12 Manufacture of tobacco products, Division 13 Manufacture of textiles, Division 26 Manufacture of computer, electronic and optical products, Division 27 Manufacture of electrical equipment, Division 29 Manufacture of motor vehicles, trailers and semi-trailers—automotive industry, Division 30 Manufacture of other transport equipment, Division 21 Manufacture of pharmaceuticals, medicinal chemical and botanical products, Division 25 Manufacture of fabricated metal products, except machinery and equipment) and Construction (Section F). According to the Global Industry Classification Standard [112], these industries would belong to the following industrial sectors: Information Technology (Sector 45: Industry group 4520 Technology Hardware & Equipment, Industry No. 452,030 Electronic Equipment, Instruments & Components), Consumer Staples (Sector 30: Industry group 3020 Food, Beverage & Tobacco, Industry No. 302,020 Food Products and No. 302,030 Tobacco), Health Care (Sector 35: Industry group 3520 Pharmaceuticals, Biotechnology & Life Sciences, Industry No. 352,020 Pharmaceuticals), Consumer Discretionary (Sector 25: Industry group 2520 Consumer Durables & Apparel, Industry No. 252,030 Textiles, Apparel & luxury goods), Materials Industry (Sector 15: group 1510 Materials for steel industry) and Industrials (Sector 20: Industry group 2010 Capitals goods for automotive and constructions).

The model presented in this paper is different from other models in the aspect of real-world-like facilities simulation and computation. The PRNG application is an important input to the research presented in the paper. This input is a crucial issue concerning the development of simulation models which correspond to actual conditions in real-life logistics facilities. Without such actual conditions, especially in the case of hypothetical systems, an analysis of the sensitivity of system parameters would not be valid (it should be noted that the model has not been validated since it is a model of the hypothetical warehouse, however it was verified [32]; in contrast to other research papers e.g., [113], the workers and equipment are not assumed to work in modeled warehouse in a constant manner, which is due to the application of the PRNG; therefore the simulation model may be more adequate for reflecting real-life situations and, at the same time, more relevant to actual working conditions). The original PRNG was used to generate picking lists with randomly selected numbers of rows and quantities of items to pick. This ensures building a hypothetical, yet close to reality, order picking process, in terms of unpredictable customers’ orders. Use of this PRNG also ensures the reflection of the real facility conditions in a simulation model. The PRNG is connected to chaos and nonlinear dynamics, whereas most of the research on the subject assumes a unified load unit being processed in the order of the picking process. The simulation model enables process analysis with non-unified load units and with real-world picking lists. In addition, the sensitivity analysis of chosen processes’ parameters ensures the system’s smooth functioning, from the viewpoint of order picking process reliability. The important aspect of this paper connected to the PRNG application was the execution analyses of the f(k) indicator. The value of f(k) reflects the estimated percentage of simulation time during which a mean of transport failure may occur, or a time when a means of transport is fully incapable for work. In short, it reflects the random idleness of the means of transport used in the research. The higher the f(k) value is, the more hazardous the order picking process is in relation to picking orders not realized in the allowed/assumed time. Therefore, managers are able to control the process, devices and means of transport used in the process, by analyzing the value of this indicator. They control the process in order to not gain high values of the indicator, especially higher than its critical value. As was mentioned before, the author’s previously presented research suggested that the critical value is f(k) = 30%. Execution of the model with higher values of f(k) significantly extends the total order picking process time, as can be observed based on the values given in Table 2 and Figure 2, Figure 3 and Figure 4. Again, when f(k) is equal to, or less than 30%, process implementation does not cause major problems, regardless of the *MTTR* value. In other words, changes in duration of the process do not have much negative impact on its implementation, while f(k) increases up to about 30%—above this value, decision-making managers might decide that one particular aisle in a warehouse should be operated by several redundant devices. Nevertheless, this would be very difficult in technological and organizational terms (in the case of e.g., use of an electric three-way stock-picker lift-truck discussed in this research), due to narrow aisles. It could be practically impossible to introduce redundant equipment (in the case of rack stacker cranes or an automated storage and retrieval system AS/RS), because of the fact that one rack stacker crane per aisle is applied to operation in a warehouse. Theoretically, it is possible to assign one rack stacker crane in order to work in several aisles; nevertheless, changes of allocation from one aisle, in which a certain stacker crane operates, to another one is not applied in actual logistics systems. This is because of the complex technological systems, the potential need for wide cross aisles at both ends of a storage zone, and long set-up times for such a device [31]). Based on the results of the model considered in this research, certain decision-making managers may also decide whether a device or mean of transport is suitable for further operation in said technological process after repairs, or whether it should be disposed of and replaced with a new device. It is worth noting that other research showed that a 10% increase of the f(k) values results in a decrease of half of the mean time to failure and mean time before failure values (results of this research are discussed in greater detail in [114]).

The limitations of the simulation model are as follows. In the model, one mean of transport and one aisle are taken into consideration, therefore for future research, the full area will be taken into consideration for the presented model. In accordance to boundary values of the parameters $p_{ij}$ and $w_{j}$, it can be stressed that higher values of these parameters can be used for analyses, since it is possible that these higher values affect the results—on the other hand, the boundary values were selected to reflect the actual (real-life) processes in order picking areas of a warehouse. Moreover, the limitation of the second of these parameters is that the parameter $w_{j}$ is indirectly random, i.e., via the randomness of the parameter $p_{ij}$. For future research, the PRNG might generate values of $w_{j}$ as well. At last, when mentioning limitations, the author suggests the uniform distribution in order to generate $p_{ij}^{*}$. Moreover, for future research, the author is considering analyses of the availability of the system, devices and means of transport.

The research potential of the issues discussed in this paper is much wider. In the future research, it is important to validate obtained results in an existing logistics facility and enriching the sensitivity analysis with changes of other parameters, as well as using software other than Plant Simulation, especially in order to compare the results. In forthcoming research, the author will reshape the simulation model in order to analyze the use of autonomous mobile robots and autonomous guided vehicles as, e.g., in [115]. It will also be profitable to transfer simulation methodology presented in this paper to other logistics facilities, e.g., freight terminals, since activity planning and optimization in these facilities can be realized by such simulation methods [116,117].

## Figures and Tables

**Table 1:** Selected ways of picking lists generating identified in the literature.

Way of Picking List Application	References that Mention Particular Way	Necessary Comments
generalized random picking lists	[19,24,34,35,36,37,38,39,40,41,42,43,44,45,46,47,48,49,50,51,52]	Fumi et al. (2013) [36] mentioned the variable picking list. Le-Duc and de Koster (2007) [24] applied random picking lists which consisted of only one line. Pawlewski (2015) [41] defined the methodology of the simulation model building, while implementing the design step of creating examples of picking lists (random or historical). Quader et al. (2016) [19] used a fixed and random picking list. Urzuà et al. (2019) [46] applied a random picking list based on historical data.
uniform distribution picking lists	[53,54,55,56,57,58,59,60,61,62,63,64,65,66,67,68,69,70,71,72,73,74,75,76,77]	Giannikas et al. (2017) [58] mentioned the uniform demand for the stock keeping unit. Lee et al. (2020) [65] applied uniform distribution picking list indirectly by implementation of uniform pick-up time. In the case of Žulj et al. (2018) [77], picking lists were indirectly connected to uniform distribution.
picking lists based on historic data	[41,46,78,79,80,81,82,83,84,85,86,87,88,89,90,91,92]	Battini et al. (2016) and Battini et al. (2015) [79,80] suggested that the actual time needed to pick an item from a vertical lift tray was the average value. Burinskienė (2010) [82] mentioned the picking list data base. Gómez-Montoya et al. (2016) [84] mentioned a variable picking list connected to empirical data. Urzêa et al. (2019) [46] applied a random picking list based on historical data.
other	[93,94,95,96,97,98,99]	Cano et al. (2017) [93] applied ad hoc picking lists. Charu et al. (2018) [94] mentioned non-uniform distribution. Chen and Wu (2005) [95] applied normal distribution picking lists. Furmans et al. (2009) [96] applied lognormal distribution and suggested pick times that follow exponential distribution. In the case of Kawczyński and Aguilar-Sommar (2006) [97], the number of products per order is variable, and it is assumed to be described by exponential distribution. Tappia et al. (2019) [98] applied pick times that follow an exponential distribution. Yu and de Koster (2009) [99] applied a random picking list with Poisson order arrivals.

**Table 2:** Numbers of product $p_{ij}$ of *i*th type (from 1st type to 10th type) in *j*th experiment (described in the columns headers) per order (*j*)—the same sample is identical to that given in Figure 1.

j	{*p_j_*_1_, *p_j_*_2_, *p_j_*_3_, *p_j_*_4_, *p_j_*_5_, *p_j_*_6_, *p_j_*_7_, *p_j_*_8_, *p_j_*_9_, *p_j_*_10_}	*j*	{*p_j_*_1_, *p_j_*_2_, *p_j_*_3_, *p_j_*_4_, *p_j_*_5_, *p_j_*_6_, *p_j_*_7_, *p_j_*_8_, *p_j_*_9_, *p_j_*_10_}	*j*	{*p_j_*_1_, *p_j_*_2_, *p_j_*_3_, *p_j_*_4_, *p_j_*_5_, *p_j_*_6_, *p_j_*_7_, *p_j_*_8_, *p_j_*_9_, *p_j_*_10_}
1	{5,9,10,6,2,8,8,0,3,3}	35	{4,7,5,0,4,5,1,2,9,9}	69	{7,4,8,9,0,1,4,2,5,4}
2	{9,4,8,7,9,1,4,6,4,8}	36	{1,9,10,7,1,7,9,7,6,4}	70	{0,2,2,6,7,1,5,4,6,4}
3	{0,6,10,6,8,10,3,1,7,4}	37	{3,2,6,9,1,8,6,0,7,6}	71	{1,3,6,3,7,9,2,9,2,2}
4	{3,5,5,3,9,9,4,6,4,1}	38	{2,9,4,10,0,9,9,2,5,2}	72	{10,3,9,8,8,0,9,9,2,8}
5	{1,9,2,6,8,8,8,3,9,0}	39	{3,5,3,0,9,8,1,2,4,3}	73	{9,6,9,9,3,2,10,3,2,8}
6	{7,1,10,6,9,2,6,0,0,3}	40	{9,3,5,5,9,1,1,7,2,5}	74	{4,1,8,4,1,5,9,3,1,2}
7	{3,2,7,3,3,9,3,6,6,5}	41	{10,2,10,8,9,0,5,2,1,4}	75	{1,6,2,6,6,10,2,6,3,8}
8	{0,4,4,5,6,6,1,6,6,2}	42	{4,3,4,6,9,1,8,10,0,8}	76	{8,6,0,4,3,3,6,3,1,5}
9	{0,6,6,10,7,6,1,10,2,4}	43	{2,6,10,3,2,6,9,6,8,7}	77	{2,10,1,10,6,5,6,3,7,6}
10	{5,8,2,9,1,10,0,7,1,4}	44	{6,2,3,1,2,6,2,0,5,3}	78	{0,1,2,8,2,5,10,5,1,7}
11	{9,0,1,7,3,8,9,9,6,8}	45	{1,6,3,4,1,5,4,4,4,3}	79	{7,9,6,2,5,2,9,7,0,8}
12	{9,7,2,7,9,10,4,5,6,5}	46	{1,1,4,7,4,4,6,8,1,7}	80	{5,3,7,9,7,3,7,6,9,9}
13	{6,1,5,7,2,5,6,2,3,9}	47	{8,6,0,0,4,5,9,6,7,0}	81	{3,5,2,4,9,8,7,7,6,4}
14	{6,1,4,5,7,10,5,7,9,6}	48	{4,5,0,2,9,8,4,5,1,6}	82	{4,6,0,5,5,8,6,4,6,1}
15	{8,4,6,7,9,9,8,8,4,7}	49	{1,9,6,2,8,2,5,4,6,3}	83	{8,5,7,9,1,0,4,5,1,3}
16	{2,9,10,3,4,5,2,10,5,3}	50	{3,7,3,9,7,9,7,2,2,9}	84	{8,7,1,7,4,9,2,0,3,9}
17	{5,5,4,6,10,2,6,4,5,1}	51	{5,5,9,8,7,8,4,0,7,2}	85	{8,2,4,8,10,7,3,6,9,3}
18	{2,6,7,9,2,0,1,4,0,5}	52	{5,7,3,8,9,9,1,4,6,7}	86	{7,4,8,9,8,4,2,7,1,3}
19	{3,2,4,9,5,4,5,8,4,2}	53	{7,9,8,1,2,8,6,9,5,5}	87	{7,8,9,2,7,9,1,9,9,7}
20	{3,5,3,1,5,8,9,5,3,9}	54	{2,3,3,10,9,2,8,9,4,5}	88	{1,3,9,7,6,4,7,4,10,0}
21	{10,5,5,5,8,1,9,7,10,2}	55	{9,0,0,9,2,8,6,0,6,2}	89	{4,9,3,5,3,5,5,3,3,1}
22	{10,10,5,7,1,2,2,5,6,7}	56	{6,5,8,7,5,5,2,6,10,2}	90	{8,8,6,9,4,9,3,4,0,4}
23	{2,3,8,4,7,6,6,8,5,8}	57	{8,4,3,3,4,7,7,9,8,7}	91	{3,9,4,9,0,1,10,6,9,0}
24	{4,8,5,3,10,6,4,4,2,3}	58	{5,4,6,7,1,1,7,8,6,8}	92	{8,8,2,7,5,4,7,1,4,6}
25	{7,3,7,9,0,2,3,5,2,0}	59	{3,0,9,9,4,1,2,0,3,8}	93	{1,6,8,8,4,7,9,8,9,6}
26	{6,7,2,6,2,2,4,2,6,9}	60	{8,3,7,0,6,9,10,3,8,9}	94	{4,2,5,8,2,8,6,8,9,4}
27	{7,5,5,4,7,9,1,0,1,5}	61	{8,9,7,6,5,6,8,4,7,2}	95	{2,0,5,4,0,10,2,7,3,5}
28	{4,1,4,5,4,6,4,3,9,8}	62	{4,10,5,2,7,10,1,9,8,3}	96	{1,6,9,0,4,1,10,6,2,5}
29	{5,7,8,8,2,8,6,6,3,5}	63	{9,9,4,1,6,7,8,3,8,4}	97	{7,10,10,1,8,2,3,5,3,8}
30	{3,6,0,5,2,9,0,1,7,4}	64	{7,9,2,7,9,3,5,8,7,7}	98	{6,1,1,7,4,1,8,0,1,10}
31	{6,2,8,9,8,4,3,8,2,9}	65	{4,0,9,6,5,5,5,6,9,9}	99	{8,7,3,9,7,7,2,6,2,2}
32	{10,9,7,7,2,2,10,4,6,5}	66	{7,8,4,1,1,3,8,6,3,3}	100	{3,8,2,10,2,10,6,2,5,8}
33	{4,5,9,1,1,2,4,1,10,10}	67	{1,4,6,3,7,2,4,2,8,1}	-	-
34	{5,8,3,8,6,9,6,2,1,3}	68	{2,8,1,5,2,10,3,1,8,8}	-	-

**Table 3:** List of statistical parameters concerning the order picking process time values, according to the simulation model, determined for 100 orders in case of data sample, in the function of *f*.

*k*	f=f(k) [%]	Total Order Picking Process Time for Sample (*t*)		$\bar{t(PS)}|f\left(k\right)$ [min]	$s_{t\left(PS\right)}|f\left(k\right)$ [min]	$s_{\bar{t(PS)}}|f\left(k\right)$ [min]	$\left|\begin{array}{l} \bar{t(PS)}|f\left(k+1\right)+\\ -\bar{t(PS)}|f\left(k\right) \end{array}\right|$ [min]
		[min]	[h]				
1	0	1591.68	26.53	15.92	1.28	0.13	0.00
2	5	1635.85	27.26	16.36	1.32	0.13	0.44
3	10	1668.71	27.81	16.68	1.51	0.15	0.33
4	15	1656.41	27.61	16.56	1.33	0.13	0.12
5	20	1727.45	28.79	17.27	1.44	0.14	0.71
6	25	1727.52	28.79	17.28	1.56	0.16	0.00
7	30	1854.48	30.91	18.54	1.80	0.18	1.27
8	35	1824.97	30.42	18.25	1.64	0.16	0.30
9	40	1931.22	32.19	19.31	1.67	0.17	1.06
10	45	2033.89	33.90	20.34	1.73	0.17	1.03
11	50	2108.16	35.14	21.08	1.80	0.18	0.74
12	55	2265.92	37.77	22.66	2.03	0.20	1.58
13	60	2345.79	39.10	23.46	2.08	0.21	0.80
14	65	2663.12	44.39	26.63	2.39	0.24	3.17
15	70	2775.42	46.26	27.75	2.63	0.26	1.12
16	75	3077.35	51.29	30.77	2.93	0.29	3.02
17	80	3525.15	58.75	35.25	3.40	0.34	4.48
18	85	4011.17	66.85	40.11	3.88	0.39	4.86
19	90	5649.00	94.15	56.49	5.65	0.57	16.38
20	95	7849.27	130.82	78.49	8.90	0.89	22.00
21	99	31966.38	532.77	319.66	48.38	4.84	241.17

**Table 4:** Use of mean of transport (EKX_515k, electric three-way stock-picker lift-truck).

*k*	f=f(k) [%]	MTTR [min]	Operational [%]		Failed [%]	$\bar{t(PS)}|f\left(k\right)$ [min]	$s_{t\left(PS\right)}|f\left(k\right)$ [min]	$s_{\bar{t(PS)}}|f\left(k\right)$ [min]
			Working	Working				
22	10	1	77.54	12.42	10.04	16.84	0.94	0.09
23	10	3	78.75	11.06	10.19	17.37	1.07	0.11
24	10	9	80.19	10.61	9.20	18.01	1.01	0.10
25	10	27	76.25	14.47	9.28	18.09	0.77	0.08
26	10	81	81.96	9.29	8.75	16.82	0.95	0.10
27	10	243	67.74	17.48	14.78	16.66	0.98	0.10
28	20	1	62.38	17.45	20.17	17.50	0.79	0.08
29	20	3	60.51	19.26	20.23	17.93	0.77	0.08
30	20	9	60.60	18.34	21.06	19.14	1.57	0.16
31	20	27	63.28	18.02	18.70	19.73	1.16	0.12
32	20	81	65.00	16.94	18.06	19.30	1.73	0.17
33	20	243	58.13	21.02	20.85	16.66	0.98	0.10
34	30	1	47.12	22.65	30.23	18.65	0.53	0.05
35	30	3	46.33	22.80	30.87	18.92	1.47	0.15
36	30	9	45.63	23.28	31.09	20.27	2.15	0.22
37	30	27	51.07	22.30	26.63	20.54	1.80	0.18
38	30	81	52.40	21.68	25.92	22.15	2.48	0.25
39	30	243	55.40	21.41	23.19	18.61	2.49	0.25
